# A first integrative study of the identity and origins of the British Dwarf Pill Millipede populations, Trachysphaera cf. lobata (Diplopoda, Glomerida, Glomeridae)

**DOI:** 10.3897/BDJ.3.e5176

**Published:** 2015-06-09

**Authors:** Jeanne Wilbrandt, Paul Lee, Helen Read, Thomas Wesener

**Affiliations:** ‡ Zoological Research Museum Koenig, Bonn, Germany; § British Myriapod & Isopod Group, Bucks, United Kingdom; | Hymettus, Midhurst, United Kingdom

**Keywords:** Integrative study, barcoding, taxonomic characters, SEM, Glomerida

## Abstract

Three populations of the pill millipede genus *Trachysphaera* Heller 1858 are present in Great Britain, one on the Isle of Wight, one in South Wales and one in mid-Wales. To identify and characterize the British *Trachysphaera* populations, the intraspecific and interspecific variation of the populations in South Wales and on the Isle of Wight were studied and evaluated in a first integrative study of members of *Trachysphaera*, utilizing barcoding and SEM. DNA was extracted from 28 British *Trachysphaera* and 10 French *T. lobata* (Ribaut 1954) specimens, one each of French T. cf. drescoi (Conde and Demange 1961) and *T. pyrenaica* (Ribaut 1908), and one of Spanish T. cf. rousseti (Demange 1959); the barcoding fragment of the COI gene was amplified and their genetic intra- and interpopulation distances compared with one another using two Italian *T.* spp. and one Croatian *T. schmidti* Heller 1858 specimens as near outgroups. To compare the genetic distances with the morphological characters, 15 characters of a total of 13 British *Trachysphaera*, together with two specimens of *T. pyrenaica*, two T. cf. drescoi and one of T. cf. rousseti were imaged, using the same individuals utilized for DNA extraction. Albeit both British populations are genetically distant, they are closely related (1.9–2.5% p-distance) to French *T. lobata*, corroborating results of earlier studies. Between different *Trachysphaera* species, genetic distance was high (16.7–18.8%). The morphological study showed the non-reliability of key taxonomic characters in *Trachysphaera*, with genetically identical individuals exhibiting morphological variation, especially on the telopods. The only observed morphological characters constant within and different between species were the number of rows of sclerotized bacilli on the tergites, as well as the shape of the male and female anal shield. Both, barcoding and the morphological study identify the British *Trachysphaera* populations as *T. lobata*.

## Introduction

Some of the most ornamented members of the pill millipedes (order Glomerida) are the dwarf species of the genus *Trachysphaera* Heller 1858, whose members possess a strongly modified appearance, resembling tiny, calcareous stones rather than animals (Fig. 1). *Trachysphaera*, sometimes still known under the synonym *Gervaisia*, is with ca. 30 described species currently the third most-diverse genus of the order Glomerida. Species of the genus show a patchy distribution, mainly in Europe, from Spain through the Caucasus and reaching their northernmost points of distribution in Germany and Poland (Tabaracu 1988, Golovatch 1989, Golovatch 2008, Kime and Enghoff 2011). Additionally, some species occur in Turkey and westernmost Iran (Golovatch 1989).

The position of *Trachysphaera* vis-à-vis other Glomerida is uncertain, with several authors putting the genus into a family of its own, the Trachysphaeridae. This placement is based on the fusion of the last tergite with the anal shield, reduction of defense glands, an unusual technique of rolling up, circular grooves on the thoracic shield, and other external peculiarities (Attems 1926, Verhoeff 1932, Schubart 1934, Hoffmann 1979). Contrastingly, the most recent classification is based exclusively on the male copulation appendages (telopods) and places them into the large subfamily Doderiinae of the Glomeridae (Mauriès 2005). The correct position of the genus can probably not be clarified until the phylogeny of the whole order is analyzed.

Even more problematic than the position of *Trachysphaera* within Glomerida is the alpha-taxonomy of the genus. Species were first described based on their peculiar external morphology alone (Verhoeff 1906, Verhoeff 1908). Once larger series of specimens were collected, these characters were discovered to be highly variable within species and even within populations. Later, researchers used the male copulation appendages, the telopods, to demarcate species (Attems 1943). However, telopods in pill millipedes are only used to hold the female, not to transfer sperm (Haacker 1964), which makes them less species-specific than in other millipedes (Mauriès 1971). The telopods were discovered to be highly variable intraspecifically, while at the same time being very similar in almost all species of *Trachysphaera* (Tabaracu 1988). The taxonomic characters used to separate *Trachysphaera* species from one another (see Tabaracu 1987) are currently ambiguous. This very difficult situation has brought despair to almost all researchers who ever worked on the taxonomy of the group (see Attems 1943, Thaler and Neuherz 1978, Tabaracu 1988, Golovatch 1989, Golovatch 2008).

Finding and collecting *Trachysphaera* specimens constitute further difficulties, since species are small, cryptic, and rare. Their dwarf habitus makes them difficult to find for a generalist researcher, as specimens are <5 mm long when walking and form a sphere with a diameter of 2–3 mm when rolled up. Discovery is also hampered by their crypsis: specimens do not move for up to 1h when disturbed (T. Wesener, personal observation), and resemble a tiny pebble of calcareous stone. Additionally, while some Austrian species are now known from a relatively wide area (Gruber 2009), most species show a very patchy distribution, being recorded only few times from less than a handful of localities (Kime and Enghoff 2011). Even where they are known to occur, we were only able to find them in a few square meters of an otherwise perfectly suitable looking habitat (T. Wesener, personal observation) or in even smaller pockets of humus rich, sandy soil within a wider area of clay (Lee et al. 2005).

Adding to these morphological peculiarities, sequencing of *Trachysphaera* specimens seems to be difficult as well. Of the 10 specimens analyzed during the 'Fauna Bavarica' project and sequenced at the Barcode of Life facility in Guelph, none yielded sequence data (Spelda et al. 2011). Apparently, DNA extraction from these tiny, heavily calcified, parasite infested specimens is challenging.

In 1984, an isolated population of *Trachysphaera* was discovered at Bembridge on the Isle of Wight, England. The species was tentatively determined as *T. lobata* (Ribaut 1954), but no male specimens were collected (Jones and Keay 1986). *T. lobata* is a species known from SE France (Kime and Enghoff 2011) and is difficult to distinguish from four other little-known *Trachysphaera* species occurring along the Spanish-French border, in particular the very similar *T. pyrenaica* (Ribaut 1907), as well as *T. rousseti* (Demange 1959), and *T. drescoi* (Conde and Demange 1961). The discovery of males within the population on the Isle of Wight (Lee et al. 2005​) and of additional *Trachysphaera* populations of unknown identity in southern and mid-Wales (Harper 2010) raised hopes of achieving deeper insights into the history and identity of the *Trachysphaera* populations in Great Britain, some of the northernmost occurrences of the genus.

For a comprehensive analysis and evaluation of the British *Trachysphaera* populations we chose an integrative (molecular and morphological) approach, applied for the first time in pill millipedes. The analysis of morphology and sequence data from the same specimen allows a direct comparison of morphological and genetic variation. With this approach we aimed to answer the question of the origin of the two British *Trachysphaera* populations, and to discover whether the two disjunct populations resembled relics or more recent (potentially anthropogenic) introductions. We also evaluated the intrapopulational, intraspecific and interspecific differences of 15 sexual and non-sexual morphological characters apart from the telopods in order to advance future taxonomic classifications of the more than 30 *Trachysphaera* species.

## Materials and methods

### Specimen selection

To investigate the morphological and molecular diversity of the British *Trachysphaera*, 28 specimens from Wales and the Isle of Wight were selected. From the mainland population of *T. lobata*, 10 specimens from a French population (Génis) were used. Additional specimens were included from the northern French-Spanish border region from three sites: (1) one male and female of *T. pyrenaica* from Grotte de l'Estellas, France, (2) one female of T. cf. rousseti from Leitza, Spain, (3) one male and female of T. cf. drescoi from Sare, France.

As near outgroups, two female specimens of undetermined Italian *Trachysphaera*, as well as a specimen of the eastern central European species and type of the genus, *T. schmidti* Heller 1858, were selected because of their divergent morphology not related to the British populations. Two specimens of the British *Polyzonium germanicum* Brandt 1837 (order Polyzoniida) were selected as far outgroups (NCBI GenBank accession numbers: KJ408478, KJ408479).

### Collecting and preservation

All animals were collected by hand. Two slightly different preservation methods were utilized: (1) Immediately after capture, specimens were put into 1.8 ml screw top vials with 98% ethanol; British *Trachysphaera* were stored in individual tubes, while all French *T. lobata* and all Italian *Trachysphaera* specimens were each put together into a single vial. (2) The specimens of *T. pyrenaica*, T. cf. rousseti and T. cf. drescoi were captured and stored in 80% ethanol, before being transferred to 98% ethanol >12 months later.

### Multi-layer photography

Before the disintegration of the specimens for the removal of tissue and SEM preparations, colored multi-layer photographs of the enrolled specimens were taken under a Leica Z6 Imaging-System. A total of 11 specimens from the Isle of Wight population, and 8 from the Welsh population were photographed. For optimal depth of field, the 10-15 single photographs taken from each specimen were put together into one multi-layer photograph using the software Auto-Montage.

### DNA extraction protocol

The tiny size of the specimens made DNA extraction difficult. In at least one case, the animal was filled with a long and massive nematomorph >4x the length of the host specimen. Due to the possible presence of parasites and the fact that no sequences of the genus (or family) were present on NCBI GenBank for comparison, no whole body extraction was utilized, but every specimen was carefully dissected. Intersegmental muscle tissue was chosen as the extraction target. Due to the small size of the specimens, the specimen was pulled apart along the tergite margins and divided into several parts, where the muscle tissue binding the tergites could be removed with fine forceps. Dissected muscle tissue was washed in a dish of ethanol to remove attached particles of the intestine, which are usually filled with cephaline gregarines.

The muscle tissue was processed with a DNAeasy Blood & Tissue kit from Qiagen following the manufacturer’s extraction protocol, except that two times 50 µl elution buffer were used to heighten the DNA yield of the extraction. DNA was extracted from a total of 44 specimens: 13 specimens of T. cf. lobata from the Isle of Wight and 15 from Wales; 10 specimens of *T. lobata* from Génis, France; one specimen of *T. pyrenaica* from the Grotte de L'Estellas; one specimen of T. cf. rousseti from Leitza, Spain; one specimen of T. cf. drescoi from Sare, France; one specimen of *T. schmidti* from Croatia and two specimens of the Italian *Trachysphaera*. The dissected specimens were also used for the SEM study (see below), while the remaining parts are conserved as voucher specimens at the ZFMK. Genomic DNA is archived in Qiagen extraction buffer and stored at -20°C at the ZFMK.

### PCR and sequencing

To gain insight into the genetic diversity of the British *Trachysphaera* populations as well as their distance to the French and Spanish taxa, the standard barcoding fragment of the cytochrome *c* oxidase subunit I (COI), a mitochondrial gene, was chosen as a marker. The COI gene was amplified using polymerase chain reaction (PCR) (Saiki et al. 1988) utilizing the HCO/LCO primer pair (LCO-1490, HCO-2198, Folmer et al. 1994), which corresponds to the COI region 1057–1500. When amplification was unsuccessful, as was the case for the *T. lobata* specimens from Génis, the Nancy & LCO primer pair was used (Simon et al. 1994). Each reaction volume (20 µl) included 5 µl of DNA extract (since the usual amounts of 1 µl and 2.5 µl did only yield weak bands), 1.3 µl dH water, 1 µl Q-solution (Qiagen), 9.5 µl Qiagen Multiplex Mix, and 1.6 µl (10 pmol/µl) of each primer. PCRs were run with a positive and a negative control. All reactions were procured with a 'touch down' PCR containing an initial denaturation at 95°C for 15 minutes, 15 (25) cycles of 94°C for 35s, 55°C (40°C) for 90s, 72°C for 90s, and a final elongation at 72°C for 10 minutes. PCRs from 34 specimens of *T. lobata* and the Italian *Trachysphaera* yielded bands, while those for *T. pyrenaica*, T. cf. rousseti and T. cf. drescoi were unsuccessful.

Purified PCR products from 34 specimens were outsourced for double-strand sequencing to a contract sequencing facility (Macrogen, Seoul, Korea) on an ABI3730 XL automatic DNA sequencer, using the same primer sets as for PCR. Sequences of a total of 27 specimens (13x Isle of Wight, 12x Wales, 2x Italy) could be obtained, while the sequences for the French *T. lobata* only contained various contaminations. Overall, PCR and sequencing success was limited (Table 1). We successfully resequenced two tissue samples (Myr924 & 925) of the French *T. lobata* as well as the *T. schmidti* sample at BGI (Beijing, China), again on an ABI3730 sequencer (primers: (S0326-0317F)D3A, (S0326-0317R)D3B). The reaction volume (5µl) included 3µl of DNA extract, 1µl of primer (3 pM), 0.5 µl BigDye, and 0.5 µl dH water. The same attempt was unsuccessful for the *T. pyrenaica* specimen.

Sequencing reads were assembled with Bioedit 7.1.3. (Hall 1999), while the identity (i.e., COI) of all sequences was confirmed with BLAST searches (Altschul et al. 1997) against the NCBI non-redundant database.

### Phylogeny reconstruction from sequence data

The analysis involved 32 nucleotide sequences (COI from 32 specimens), with a total of 660 positions in the final dataset. The number of base substitutions per site from between sequences were determined using MEGA (v. 5.2, Tamura et al. 2011). The phylogeny was inferred by using the Maximum Likelihood method based on the Tamura 3-parameter model (Tamura 1992). Initial tree(s) for the heuristic search were obtained automatically by applying Neighbor-Join and BioNJ algorithms to a matrix of pairwise distances estimated using the Maximum Composite Likelihood (MCL) approach, and then selecting the topology with superior log likelihood value. A discrete Gamma distribution was used to model evolutionary rate differences among sites (5 categories (+*G*, parameter = 0.7759)). The nucleotide frequencies A = 0.3273, T = 0.3273, C = 0.1727, G = 0.1727. Codon positions included were 1st+2nd+3rd+Non-coding, while all positions containing gaps and missing data were eliminated.

The tree with the highest log likelihood (-2177.4216) is shown below (Analysis section). The percentage of trees in which the associated taxa clustered together is shown next to the branches (bootstrap). The tree is drawn to scale, with branch lengths reflecting the number of substitutions per site.

### Scanning electron microscopy and morphological character analysis

To evaluate the intra- and interspecific variation of closely related *Trachysphaera* species, a total of 19 specimens (3 males, 2 females from Isle of Wight; 1 male, 7 females from Wales; 1 male, 3 females of *T. pyrenaica*; 1 male, 1 female T. cf. drescoi) of *Trachysphaera*, all of which were also subject to DNA extraction, were studied using scanning electron microscopy. Objects prepared for SEM were: (1) anal shield; (2) anterior body part including head, collum, thoracic shield and tergite 3; (3) midbody tergite (for body part and tergite nomenclature see Fig. 2); (4) male telopods. For scanning electron microscopy, samples were cleaned manually and dehydrated in an ethanol series (80%, 90%, 95% and twice in 100%) and air-dried overnight. The samples were then mounted on aluminum stubs before being coated with gold (layer of ca. 40 nm) in a sputter coater. SEM micrographs were taken using a Hitachi S2460N SEM, based at the ZMFK. Ultrasonic cleaning of the often dirt-covered samples was not attempted, because the risk of destroying the unique surface structure of the tergites was deemed too high. Pictures of the state of all characters in all specimens (as far as they could be obtained) are available on MorphBank (Collection ID 853154, specimen IDs (MBsID) see Checklist Materials, image IDs (MBiID) are given where necessary and not fully listed (326 images in total)).

A total of 15 (11, 4 of which separately given for male and female) morphological characters commonly employed in the taxonomy of *Trachysphaera* were investigated for their taxonomic value (see Table 2). Some characters were selected because they greatly improved the taxonomy in the giant pill millipede order Sphaerotheriida (VandenSpiegel et al. 2002, Wesener 2009), such as the endotergum, i.e., the underside of the tergites. Each investigated population was scored individually (see character matrix at Table 3); when two different character states were present in the population sample (which was often the case), both character states were coded as 'U' (unresolved). In another step, the telopods of *T. lobata* populations from Wales and the Isle of Wight, *T. pyrenaica* and T. cf. drescoi were compared. Note that not all specimens used in this study were analyzed with all used techniques.

## Checklists

### Checklist of Glomeridae used in this study

#### Trachysphaera lobata

(Ribaut 1954)

http://www.faunaeur.org/full_results.php?id=326679

##### Materials

- **Type status:** Other material. **Occurrence:** catalogNumber: 890; recordedBy: H. Read; individualID: TW01; individualCount: 1; sex: female; lifeStage: adult; **Taxon:** scientificName: Trachysphaera lobata; phylum: Arthropoda; class: Diplopoda; order: Glomerida; family: Glomeridae; genus: Trachysphaera; taxonRank: species; scientificNameAuthorship: (Ribaut 1954); **Location:** locationID: 1; country: Great Britain; stateProvince: South East England; locality: Isle of Wight, coast at Bembridge, western end of site; **Event:** eventDate: 22.ii.2011; year: 2011; month: 2; day: 22; **Record Level:** institutionID: ZFMK; collectionID: MYR
- **Type status:** Other material. **Occurrence:** catalogNumber: 891; recordedBy: P. Lee, S. Gregory, H. Read; individualID: TW02; individualCount: 1; sex: female; lifeStage: adult; **Taxon:** scientificName: Trachysphaera *lobata*; phylum: Arthropoda; class: Diplopoda; order: Glomerida; family: Glomeridae; genus: Trachysphaera; taxonRank: species; scientificNameAuthorship: (Ribaut 1954); **Location:** locationID: 1; country: Great Britain; stateProvince: South East England; locality: Isle of Wight, coast at Bembridge, main area; **Event:** eventDate: 22.ii.2011; year: 2011; month: 2; day: 22; **Record Level:** institutionID: ZFMK; collectionID: MYR
- **Type status:** Other material. **Occurrence:** catalogNumber: 892; recordedBy: P. Lee, S. Gregory, H. Read; individualID: TW03; individualCount: 1; sex: female; lifeStage: adult; **Taxon:** scientificName: Trachysphaera *lobata*; phylum: Arthropoda; class: Diplopoda; order: Glomerida; family: Glomeridae; genus: Trachysphaera; taxonRank: species; scientificNameAuthorship: (Ribaut 1954); **Location:** locationID: 1; country: Great Britain; stateProvince: South East England; locality: Isle of Wight, coast at Bembridge, main area; **Event:** eventDate: 22.ii.2011; year: 2011; month: 2; day: 22; **Record Level:** institutionID: ZFMK; collectionID: MYR
- **Type status:** Other material. **Occurrence:** catalogNumber: 893; recordedBy: I. Morgan; individualID: TW04; individualCount: 1; sex: female; lifeStage: adult; **Taxon:** scientificName: Trachysphaera *lobata*; phylum: Arthropoda; class: Diplopoda; order: Glomerida; family: Glomeridae; genus: Trachysphaera; taxonRank: species; scientificNameAuthorship: (Ribaut 1954); **Location:** locationID: 2; country: Great Britain; stateProvince: Wales; locality: South of Berwick, Bynea, Llanelli, Carmarthenshire; **Event:** eventDate: iii.2011; year: 2011; month: 3; **Record Level:** institutionID: ZFMK; collectionID: MYR
- **Type status:** Other material. **Occurrence:** catalogNumber: 894; recordedBy: I. Morgan; individualID: TW05; individualCount: 1; sex: female; lifeStage: adult; **Taxon:** scientificName: Trachysphaera *lobata*; phylum: Arthropoda; class: Diplopoda; order: Glomerida; family: Glomeridae; genus: Trachysphaera; taxonRank: species; scientificNameAuthorship: (Ribaut 1954); **Location:** locationID: 2; country: Great Britain; stateProvince: Wales; locality: South of Berwick, Bynea, Llanelli, Carmarthenshire; **Event:** eventDate: iii.2011; year: 2011; month: 3; **Record Level:** institutionID: ZFMK; collectionID: MYR
- **Type status:** Other material. **Occurrence:** catalogNumber: 897; recordedBy: H. Read; individualID: TW11; individualCount: 1; sex: female; lifeStage: adult; **Taxon:** scientificName: Trachysphaera *lobata*; phylum: Arthropoda; class: Diplopoda; order: Glomerida; family: Glomeridae; genus: Trachysphaera; taxonRank: species; scientificNameAuthorship: (Ribaut 1954); **Location:** locationID: 1; country: Great Britain; stateProvince: South East England; locality: Isle of Wight, coast at Bembridge, western end of site; **Event:** eventDate: 22.ii.2011; year: 2011; month: 2; day: 22; **Record Level:** institutionID: ZFMK; collectionID: MYR
- **Type status:** Other material. **Occurrence:** catalogNumber: 898; recordedBy: P. Lee, S. Gregory, H. Read; individualID: TW12; individualCount: 1; sex: male; lifeStage: adult; **Taxon:** scientificName: Trachysphaera *lobata*; phylum: Arthropoda; class: Diplopoda; order: Glomerida; family: Glomeridae; genus: Trachysphaera; taxonRank: species; scientificNameAuthorship: (Ribaut 1954); **Location:** locationID: 1; country: Great Britain; stateProvince: South East England; locality: Isle of Wight, coast at Bembridge, main area; **Event:** eventDate: 22.ii.2011; year: 2011; month: 2; day: 22; **Record Level:** institutionID: ZFMK; collectionID: MYR
- **Type status:** Other material. **Occurrence:** catalogNumber: 899; recordedBy: P. Lee, S. Gregory, H. Read; individualID: TW13; individualCount: 1; sex: female; lifeStage: adult; **Taxon:** scientificName: Trachysphaera *lobata*; phylum: Arthropoda; class: Diplopoda; order: Glomerida; family: Glomeridae; genus: Trachysphaera; taxonRank: species; scientificNameAuthorship: (Ribaut 1954); **Location:** locationID: 1; country: Great Britain; stateProvince: South East England; locality: Isle of Wight, coast at Bembridge, main area; **Event:** eventDate: 22.ii.2011; year: 2011; month: 2; day: 22; **Record Level:** institutionID: ZFMK; collectionID: MYR
- **Type status:** Other material. **Occurrence:** catalogNumber: 900; recordedBy: P. Lee, S. Gregory, H. Read; individualID: TW14; individualCount: 1; sex: male; lifeStage: adult; **Taxon:** scientificName: Trachysphaera *lobata*; phylum: Arthropoda; class: Diplopoda; order: Glomerida; family: Glomeridae; genus: Trachysphaera; taxonRank: species; scientificNameAuthorship: (Ribaut 1954); **Location:** locationID: 1; country: Great Britain; stateProvince: South East England; locality: Isle of Wight, coast at Bembridge, main area; **Event:** eventDate: 22.ii.2011; year: 2011; month: 2; day: 22; **Record Level:** institutionID: ZFMK; collectionID: MYR
- **Type status:** Other material. **Occurrence:** catalogNumber: 901; recordedBy: P. Lee, S. Gregory, H. Read; individualID: TW15; individualCount: 1; sex: male; lifeStage: adult; **Taxon:** scientificName: Trachysphaera *lobata*; phylum: Arthropoda; class: Diplopoda; order: Glomerida; family: Glomeridae; genus: Trachysphaera; taxonRank: species; scientificNameAuthorship: (Ribaut 1954); **Location:** locationID: 1; country: Great Britain; stateProvince: South East England; locality: Isle of Wight, coast at Bembridge, main area; **Event:** eventDate: 22.ii.2011; year: 2011; month: 2; day: 22; **Record Level:** institutionID: ZFMK; collectionID: MYR
- **Type status:** Other material. **Occurrence:** catalogNumber: 902; recordedBy: P. Lee, S. Gregory, H. Read; individualID: TW16; individualCount: 1; sex: male; lifeStage: adult; **Taxon:** scientificName: Trachysphaera *lobata*; phylum: Arthropoda; class: Diplopoda; order: Glomerida; family: Glomeridae; genus: Trachysphaera; taxonRank: species; scientificNameAuthorship: (Ribaut 1954); **Location:** locationID: 1; country: Great Britain; stateProvince: South East England; locality: Isle of Wight, coast at Bembridge, main area; **Event:** eventDate: 22.ii.2011; year: 2011; month: 2; day: 22; **Record Level:** institutionID: ZFMK; collectionID: MYR
- **Type status:** Other material. **Occurrence:** catalogNumber: 903; recordedBy: P. Lee, S. Gregory, H. Read; individualID: TW17; individualCount: 1; sex: male; lifeStage: adult; **Taxon:** scientificName: Trachysphaera *lobata*; phylum: Arthropoda; class: Diplopoda; order: Glomerida; family: Glomeridae; genus: Trachysphaera; taxonRank: species; scientificNameAuthorship: (Ribaut 1954); **Location:** locationID: 1; country: Great Britain; stateProvince: South East England; locality: Isle of Wight, coast at Bembridge, main area; **Event:** eventDate: 22.ii.2011; year: 2011; month: 2; day: 22; **Record Level:** institutionID: ZFMK; collectionID: MYR
- **Type status:** Other material. **Occurrence:** catalogNumber: 904; recordedBy: P. Lee, S. Gregory, H. Read; individualID: TW18; individualCount: 1; sex: female; lifeStage: adult; **Taxon:** scientificName: Trachysphaera *lobata*; phylum: Arthropoda; class: Diplopoda; order: Glomerida; family: Glomeridae; genus: Trachysphaera; taxonRank: species; scientificNameAuthorship: (Ribaut 1954); **Location:** locationID: 1; country: Great Britain; stateProvince: South East England; locality: Isle of Wight, coast at Bembridge, main area; **Event:** eventDate: 22.ii.2011; year: 2011; month: 2; day: 22; **Record Level:** institutionID: ZFMK; collectionID: MYR
- **Type status:** Other material. **Occurrence:** catalogNumber: 905; recordedBy: P. Lee, S. Gregory, H. Read; individualID: TW19; individualCount: 1; sex: male; lifeStage: adult; **Taxon:** scientificName: Trachysphaera *lobata*; phylum: Arthropoda; class: Diplopoda; order: Glomerida; family: Glomeridae; genus: Trachysphaera; taxonRank: species; scientificNameAuthorship: (Ribaut 1954); **Location:** locationID: 1; country: Great Britain; stateProvince: South East England; locality: Isle of Wight, coast at Bembridge, main area; **Event:** eventDate: 22.ii.2011; year: 2011; month: 2; day: 22; **Record Level:** institutionID: ZFMK; collectionID: MYR
- **Type status:** Other material. **Occurrence:** catalogNumber: 906; recordedBy: P. Lee, S. Gregory, H. Read; individualID: TW20; individualCount: 1; sex: male; lifeStage: adult; **Taxon:** scientificName: Trachysphaera *lobata*; phylum: Arthropoda; class: Diplopoda; order: Glomerida; family: Glomeridae; genus: Trachysphaera; taxonRank: species; scientificNameAuthorship: (Ribaut 1954); **Location:** locationID: 1; country: Great Britain; stateProvince: South East England; locality: Isle of Wight, coast at Bembridge, main area; **Event:** eventDate: 22.ii.2011; year: 2011; month: 2; day: 22; **Record Level:** institutionID: ZFMK; collectionID: MYR
- **Type status:** Other material. **Occurrence:** catalogNumber: 907; recordedBy: I. Morgan; individualID: TW21; individualCount: 1; sex: male; lifeStage: adult; **Taxon:** scientificName: Trachysphaera *lobata*; phylum: Arthropoda; class: Diplopoda; order: Glomerida; family: Glomeridae; genus: Trachysphaera; taxonRank: species; scientificNameAuthorship: (Ribaut 1954); **Location:** locationID: 2; country: Great Britain; stateProvince: Wales; locality: South of Berwick, Bynea, Llanelli, Carmarthenshire; **Event:** eventDate: iii.2011; year: 2011; month: 3; **Record Level:** institutionID: ZFMK; collectionID: MYR
- **Type status:** Other material. **Occurrence:** catalogNumber: 908; recordedBy: I. Morgan; individualID: TW22; individualCount: 1; sex: female; lifeStage: adult; **Taxon:** scientificName: Trachysphaera *lobata*; phylum: Arthropoda; class: Diplopoda; order: Glomerida; family: Glomeridae; genus: Trachysphaera; taxonRank: species; scientificNameAuthorship: (Ribaut 1954); **Location:** locationID: 2; country: Great Britain; stateProvince: Wales; locality: South of Berwick, Bynea, Llanelli, Carmarthenshire; **Event:** eventDate: iii.2011; year: 2011; month: 3; **Record Level:** institutionID: ZFMK; collectionID: MYR
- **Type status:** Other material. **Occurrence:** catalogNumber: 909; recordedBy: I. Morgan; individualID: TW23; individualCount: 1; sex: female; lifeStage: adult; **Taxon:** scientificName: Trachysphaera *lobata*; phylum: Arthropoda; class: Diplopoda; order: Glomerida; family: Glomeridae; genus: Trachysphaera; taxonRank: species; scientificNameAuthorship: (Ribaut 1954); **Location:** locationID: 2; country: Great Britain; stateProvince: Wales; locality: South of Berwick, Bynea, Llanelli, Carmarthenshire; **Event:** eventDate: iii.2011; year: 2011; month: 3; **Record Level:** institutionID: ZFMK; collectionID: MYR
- **Type status:** Other material. **Occurrence:** catalogNumber: 910; recordedBy: I. Morgan; individualID: TW24; individualCount: 1; sex: female; lifeStage: adult; **Taxon:** scientificName: Trachysphaera *lobata*; phylum: Arthropoda; class: Diplopoda; order: Glomerida; family: Glomeridae; genus: Trachysphaera; taxonRank: species; scientificNameAuthorship: (Ribaut 1954); **Location:** locationID: 2; country: Great Britain; stateProvince: Wales; locality: South of Berwick, Bynea, Llanelli, Carmarthenshire; **Event:** eventDate: iii.2011; year: 2011; month: 3; **Record Level:** institutionID: ZFMK; collectionID: MYR
- **Type status:** Other material. **Occurrence:** catalogNumber: 911; recordedBy: I. Morgan; individualID: TW25; individualCount: 1; sex: female; lifeStage: adult; **Taxon:** scientificName: Trachysphaera *lobata*; phylum: Arthropoda; class: Diplopoda; order: Glomerida; family: Glomeridae; genus: Trachysphaera; taxonRank: species; scientificNameAuthorship: (Ribaut 1954); **Location:** locationID: 2; country: Great Britain; stateProvince: Wales; locality: South of Berwick, Bynea, Llanelli, Carmarthenshire; **Event:** eventDate: iii.2011; year: 2011; month: 3; **Record Level:** institutionID: ZFMK; collectionID: MYR
- **Type status:** Other material. **Occurrence:** catalogNumber: 912; recordedBy: I. Morgan; individualID: TW26; individualCount: 1; sex: female; lifeStage: adult; **Taxon:** scientificName: Trachysphaera *lobata*; phylum: Arthropoda; class: Diplopoda; order: Glomerida; family: Glomeridae; genus: Trachysphaera; taxonRank: species; scientificNameAuthorship: (Ribaut 1954); **Location:** locationID: 2; country: Great Britain; stateProvince: Wales; locality: South of Berwick, Bynea, Llanelli, Carmarthenshire; **Event:** eventDate: iii.2011; year: 2011; month: 3; **Record Level:** institutionID: ZFMK; collectionID: MYR
- **Type status:** Other material. **Occurrence:** catalogNumber: 913; recordedBy: I. Morgan; individualID: TW27; individualCount: 1; sex: female; lifeStage: adult; **Taxon:** scientificName: Trachysphaera *lobata*; phylum: Arthropoda; class: Diplopoda; order: Glomerida; family: Glomeridae; genus: Trachysphaera; taxonRank: species; scientificNameAuthorship: (Ribaut 1954); **Location:** locationID: 2; country: Great Britain; stateProvince: Wales; locality: South of Berwick, Bynea, Llanelli, Carmarthenshire; **Event:** eventDate: iii.2011; year: 2011; month: 3; **Record Level:** institutionID: ZFMK; collectionID: MYR
- **Type status:** Other material. **Occurrence:** catalogNumber: 914; recordedBy: I. Morgan; individualID: TW28; individualCount: 1; sex: female; lifeStage: adult; **Taxon:** scientificName: Trachysphaera *lobata*; phylum: Arthropoda; class: Diplopoda; order: Glomerida; family: Glomeridae; genus: Trachysphaera; taxonRank: species; scientificNameAuthorship: (Ribaut 1954); **Location:** locationID: 2; country: Great Britain; stateProvince: Wales; locality: South of Berwick, Bynea, Llanelli, Carmarthenshire; **Event:** eventDate: iii.2011; year: 2011; month: 3; **Record Level:** institutionID: ZFMK; collectionID: MYR
- **Type status:** Other material. **Occurrence:** catalogNumber: 915; recordedBy: I. Morgan; individualID: TW29; individualCount: 1; sex: female; lifeStage: adult; **Taxon:** scientificName: Trachysphaera *lobata*; phylum: Arthropoda; class: Diplopoda; order: Glomerida; family: Glomeridae; genus: Trachysphaera; taxonRank: species; scientificNameAuthorship: (Ribaut 1954); **Location:** locationID: 2; country: Great Britain; stateProvince: Wales; locality: South of Berwick, Bynea, Llanelli, Carmarthenshire; **Event:** eventDate: iii.2011; year: 2011; month: 3; **Record Level:** institutionID: ZFMK; collectionID: MYR
- **Type status:** Other material. **Occurrence:** catalogNumber: 916; recordedBy: I. Morgan; individualID: TW30; individualCount: 1; sex: female; lifeStage: adult; **Taxon:** scientificName: Trachysphaera *lobata*; phylum: Arthropoda; class: Diplopoda; order: Glomerida; family: Glomeridae; genus: Trachysphaera; taxonRank: species; scientificNameAuthorship: (Ribaut 1954); **Location:** locationID: 2; country: Great Britain; stateProvince: Wales; locality: South of Berwick, Bynea, Llanelli, Carmarthenshire; **Event:** eventDate: iii.2011; year: 2011; month: 3; **Record Level:** institutionID: ZFMK; collectionID: MYR
- **Type status:** Other material. **Occurrence:** catalogNumber: 917; recordedBy: I. Morgan; individualID: TW31; individualCount: 1; sex: female; lifeStage: adult; **Taxon:** scientificName: Trachysphaera *lobata*; phylum: Arthropoda; class: Diplopoda; order: Glomerida; family: Glomeridae; genus: Trachysphaera; taxonRank: species; scientificNameAuthorship: (Ribaut 1954); **Location:** locationID: 2; country: Great Britain; stateProvince: Wales; locality: South of Berwick, Bynea, Llanelli, Carmarthenshire; **Event:** eventDate: iii.2011; year: 2011; month: 3; **Record Level:** institutionID: ZFMK; collectionID: MYR
- **Type status:** Other material. **Occurrence:** catalogNumber: 918; recordedBy: I. Morgan; individualID: TW32; individualCount: 1; sex: female; lifeStage: adult; **Taxon:** scientificName: Trachysphaera *lobata*; phylum: Arthropoda; class: Diplopoda; order: Glomerida; family: Glomeridae; genus: Trachysphaera; taxonRank: species; scientificNameAuthorship: (Ribaut 1954); **Location:** locationID: 2; country: Great Britain; stateProvince: Wales; locality: South of Berwick, Bynea, Llanelli, Carmarthenshire; **Event:** eventDate: iii.2011; year: 2011; month: 3; **Record Level:** institutionID: ZFMK; collectionID: MYR
- **Type status:** Other material. **Occurrence:** catalogNumber: 919; recordedBy: I. Morgan; individualID: TW33; individualCount: 1; sex: female; lifeStage: adult; **Taxon:** scientificName: Trachysphaera *lobata*; phylum: Arthropoda; class: Diplopoda; order: Glomerida; family: Glomeridae; genus: Trachysphaera; taxonRank: species; scientificNameAuthorship: (Ribaut 1954); **Location:** locationID: 2; country: Great Britain; stateProvince: Wales; locality: South of Berwick, Bynea, Llanelli, Carmarthenshire; **Event:** eventDate: iii.2011; year: 2011; month: 3; **Record Level:** institutionID: ZFMK; collectionID: MYR
- **Type status:** Other material. **Occurrence:** catalogNumber: 920; recordedBy: Dr. Kime; individualID: TW47; individualCount: 1; sex: female; lifeStage: adult; **Taxon:** scientificName: Trachysphaera *lobata*; phylum: Arthropoda; class: Diplopoda; order: Glomerida; family: Glomeridae; genus: Trachysphaera; taxonRank: species; scientificNameAuthorship: (Ribaut 1954); **Location:** locationID: 3; country: France; stateProvince: Aquitaine; locality: Dép. Dordogne, Génis, gorge of the River Auvézère; **Event:** eventDate: viii.2011; year: 2011; month: 8; **Record Level:** institutionID: ZFMK; collectionID: MYR
- **Type status:** Other material. **Occurrence:** catalogNumber: 921; recordedBy: Dr. Kime; individualID: TW48; individualCount: 1; sex: female; lifeStage: adult; **Taxon:** scientificName: Trachysphaera *lobata*; phylum: Arthropoda; class: Diplopoda; order: Glomerida; family: Glomeridae; genus: Trachysphaera; taxonRank: species; scientificNameAuthorship: (Ribaut 1954); **Location:** locationID: 3; country: France; stateProvince: Aquitaine; locality: Dép. Dordogne, Génis, gorge of the River Auvézère; **Event:** eventDate: viii.2011; year: 2011; month: 8; **Record Level:** institutionID: ZFMK; collectionID: MYR
- **Type status:** Other material. **Occurrence:** catalogNumber: 922; recordedBy: Dr. Kime; individualID: TW49; individualCount: 1; sex: female; lifeStage: adult; **Taxon:** scientificName: Trachysphaera *lobata*; phylum: Arthropoda; class: Diplopoda; order: Glomerida; family: Glomeridae; genus: Trachysphaera; taxonRank: species; scientificNameAuthorship: (Ribaut 1954); **Location:** locationID: 3; country: France; stateProvince: Aquitaine; locality: Dép. Dordogne, Génis, gorge of the River Auvézère; **Event:** eventDate: viii.2011; year: 2011; month: 8; **Record Level:** institutionID: ZFMK; collectionID: MYR
- **Type status:** Other material. **Occurrence:** catalogNumber: 923; recordedBy: Dr. Kime; individualID: TW50; individualCount: 1; sex: female; lifeStage: adult; **Taxon:** scientificName: Trachysphaera *lobata*; phylum: Arthropoda; class: Diplopoda; order: Glomerida; family: Glomeridae; genus: Trachysphaera; taxonRank: species; scientificNameAuthorship: (Ribaut 1954); **Location:** locationID: 3; country: France; stateProvince: Aquitaine; locality: Dép. Dordogne, Génis, gorge of the River Auvézère; **Event:** eventDate: viii.2011; year: 2011; month: 8; **Record Level:** institutionID: ZFMK; collectionID: MYR
- **Type status:** Other material. **Occurrence:** catalogNumber: 924; recordedBy: Dr. Kime; individualID: TW51; individualCount: 1; sex: female; lifeStage: adult; **Taxon:** scientificName: Trachysphaera *lobata*; phylum: Arthropoda; class: Diplopoda; order: Glomerida; family: Glomeridae; genus: Trachysphaera; taxonRank: species; scientificNameAuthorship: (Ribaut 1954); **Location:** locationID: 3; country: France; stateProvince: Aquitaine; locality: Dép. Dordogne, Génis, gorge of the River Auvézère; **Event:** eventDate: viii.2011; year: 2011; month: 8; **Record Level:** institutionID: ZFMK; collectionID: MYR
- **Type status:** Other material. **Occurrence:** catalogNumber: 925; recordedBy: Dr. Kime; individualID: TW52; individualCount: 1; sex: female; lifeStage: adult; **Taxon:** scientificName: Trachysphaera *lobata*; phylum: Arthropoda; class: Diplopoda; order: Glomerida; family: Glomeridae; genus: Trachysphaera; taxonRank: species; scientificNameAuthorship: (Ribaut 1954); **Location:** locationID: 3; country: France; stateProvince: Aquitaine; locality: Dép. Dordogne, Génis, gorge of the River Auvézère; **Event:** eventDate: viii.2011; year: 2011; month: 8; **Record Level:** institutionID: ZFMK; collectionID: MYR
- **Type status:** Other material. **Occurrence:** catalogNumber: 926; recordedBy: Dr. Kime; individualID: TW53; individualCount: 1; sex: female; lifeStage: adult; **Taxon:** scientificName: Trachysphaera *lobata*; phylum: Arthropoda; class: Diplopoda; order: Glomerida; family: Glomeridae; genus: Trachysphaera; taxonRank: species; scientificNameAuthorship: (Ribaut 1954); **Location:** locationID: 3; country: France; stateProvince: Aquitaine; locality: Dép. Dordogne, Génis, gorge of the River Auvézère; **Event:** eventDate: viii.2011; year: 2011; month: 8; **Record Level:** institutionID: ZFMK; collectionID: MYR
- **Type status:** Other material. **Occurrence:** catalogNumber: 927; recordedBy: Dr. Kime; individualID: TW54; individualCount: 1; sex: female; lifeStage: adult; **Taxon:** scientificName: Trachysphaera *lobata*; phylum: Arthropoda; class: Diplopoda; order: Glomerida; family: Glomeridae; genus: Trachysphaera; taxonRank: species; scientificNameAuthorship: (Ribaut 1954); **Location:** locationID: 3; country: France; stateProvince: Aquitaine; locality: Dép. Dordogne, Génis, gorge of the River Auvézère; **Event:** eventDate: viii.2011; year: 2011; month: 8; **Record Level:** institutionID: ZFMK; collectionID: MYR
- **Type status:** Other material. **Occurrence:** catalogNumber: 928; recordedBy: Dr. Kime; individualID: TW55; individualCount: 1; sex: female; lifeStage: adult; **Taxon:** scientificName: Trachysphaera *lobata*; phylum: Arthropoda; class: Diplopoda; order: Glomerida; family: Glomeridae; genus: Trachysphaera; taxonRank: species; scientificNameAuthorship: (Ribaut 1954); **Location:** locationID: 3; country: France; stateProvince: Aquitaine; locality: Dép. Dordogne, Génis, gorge of the River Auvézère; **Event:** eventDate: viii.2011; year: 2011; month: 8; **Record Level:** institutionID: ZFMK; collectionID: MYR
- **Type status:** Other material. **Occurrence:** catalogNumber: 930; recordedBy: Dr. Kime; individualID: TW57; individualCount: 1; sex: female; lifeStage: adult; **Taxon:** scientificName: Trachysphaera *lobata*; phylum: Arthropoda; class: Diplopoda; order: Glomerida; family: Glomeridae; genus: Trachysphaera; taxonRank: species; scientificNameAuthorship: (Ribaut 1954); **Location:** locationID: 3; country: France; stateProvince: Aquitaine; locality: Dép. Dordogne, Génis, gorge of the River Auvézère; **Event:** eventDate: viii.2011; year: 2011; month: 8; **Record Level:** institutionID: ZFMK; collectionID: MYR

##### Notes

Addtional individual information in Table 4.

#### Trachysphaera pyrenaica

(Ribaut 1907)

http://www.faunaeur.org/full_results.php?id=326684

##### Materials

- **Type status:** Other material. **Occurrence:** catalogNumber: 78; recordedBy: A. Schönhofer; individualID: GEU157; individualCount: 1; sex: female; lifeStage: adult; **Taxon:** scientificName: Trachysphaera *pyrenaica*; phylum: Arthropoda; class: Diplopoda; order: Glomerida; family: Glomeridae; genus: Trachysphaera; taxonRank: species; scientificNameAuthorship: (Ribaut 1907); **Location:** locationID: 4; country: France; stateProvince: Midi-Pyrénées; locality: Dép. Ariège, NW Cazavet, sourroundings of Grotte de l’Estellas; **Event:** eventDate: 09.x.2009; year: 2009; month: 10; day: 9; **Record Level:** institutionID: ZFMK; collectionID: MYR
- **Type status:** Other material. **Occurrence:** catalogNumber: 78; recordedBy: A. Schönhofer; individualID: GEU157; individualCount: 1; sex: male; lifeStage: adult; **Taxon:** scientificName: Trachysphaera *pyrenaica*; phylum: Arthropoda; class: Diplopoda; order: Glomerida; family: Glomeridae; genus: Trachysphaera; taxonRank: species; scientificNameAuthorship: (Ribaut 1907); **Location:** locationID: 4; country: France; stateProvince: Midi-Pyrénées; locality: Dép. Ariège, NW Cazavet, sourroundings of Grotte de l’Estellas; **Event:** eventDate: 09.x.2009; year: 2009; month: 10; day: 9; **Record Level:** institutionID: ZFMK; collectionID: MYR

##### Notes

Additional individual information in Table 5.

#### Trachysphaera rousseti

(Demange 1959)

##### Materials

- **Type status:** Other material. **Occurrence:** catalogNumber: 744; recordedBy: H. Read; individualID: GL017; individualCount: 1; sex: female; lifeStage: adult; **Taxon:** scientificName: Trachysphaera cf. *rousseti*; phylum: Arthropoda; class: Diplopoda; order: Glomerida; family: Glomeridae; genus: Trachysphaera; taxonRank: species; scientificNameAuthorship: (Demange 1959); **Location:** locationID: 5; country: Spain; stateProvince: Navarra; locality: Leitza, Ariz Mendiak 'Kornieta'; **Event:** eventDate: 20.iv.2009; year: 2009; month: 4; day: 20; **Record Level:** institutionID: ZFMK; collectionID: MYR

##### Notes

Additional individual information in Table 6.

#### Trachysphaera drescoi

(Conde & Demange 1961)

http://www.faunaeur.org/full_results.php?id=326668

##### Materials

- **Type status:** Other material. **Occurrence:** catalogNumber: 80; recordedBy: A. Schönhofer; individualID: GEU158; individualCount: 1; sex: female; lifeStage: adult; **Taxon:** scientificName: Trachysphaera cf. *drescoi*; phylum: Arthropoda; class: Diplopoda; order: Glomerida; family: Glomeridae; genus: Trachysphaera; taxonRank: species; scientificNameAuthorship: (Conde & Demange 1961); **Location:** locationID: 6; country: France; stateProvince: Aquitaine; locality: Dép. Pyrénées-Atlantiques, Sare, Grand Grotte de Sare; **Event:** eventDate: 16.-17.x.2009; year: 2009; month: 10; **Record Level:** institutionID: ZFMK; collectionID: MYR
- **Type status:** Other material. **Occurrence:** catalogNumber: 80; recordedBy: A. Schönhofer; individualID: GEU158; individualCount: 1; sex: male; lifeStage: adult; **Taxon:** scientificName: Trachysphaera cf. *drescoi*; phylum: Arthropoda; class: Diplopoda; order: Glomerida; family: Glomeridae; genus: Trachysphaera; taxonRank: species; scientificNameAuthorship: (Conde & Demange 1961); **Location:** locationID: 6; country: France; stateProvince: Aquitaine; locality: Dép. Pyrénées-Atlantiques, Sare, Grand Grotte de Sare; **Event:** eventDate: 16.-17.x.2009; year: 2009; month: 10; **Record Level:** institutionID: ZFMK; collectionID: MYR

##### Notes

Additional individual information in Table 7.

#### Trachysphaera schmidti

Heller 1858

http://www.faunaeur.org/full_results.php?id=326688

##### Materials

- **Type status:** Other material. **Occurrence:** catalogNumber: 860; recordedBy: H. S. Reip; individualID: BGI-MYR-16; individualCount: 1; sex: female; lifeStage: adult; **Taxon:** scientificName: Trachysphaera *schmidti*; phylum: Arthropoda; class: Diplopoda; order: Glomerida; family: Glomeridae; genus: Trachysphaera; taxonRank: species; scientificNameAuthorship: Heller 1858; **Location:** locationID: 8; country: Croatia; locality: Rijeka, Velika Kapela; **Event:** eventDate: 15.x.2010; year: 2010; month: 10; day: 15; **Record Level:** institutionID: ZFMK; collectionID: MYR

##### Notes

Additional individual information in Table 8.

#### Trachysphaera sp.

##### Materials

- **Type status:** Other material. **Occurrence:** catalogNumber: 895; recordedBy: T. Wesener; individualID: TW06; individualCount: 1; sex: female; lifeStage: adult; **Taxon:** scientificName: Trachysphaera sp.; phylum: Arthropoda; class: Diplopoda; order: Glomerida; family: Glomeridae; genus: Trachysphaera; taxonRank: species; **Location:** locationID: 7; country: Italy; stateProvince: Piemonte; locality: Oropa, NW Sanctuary of Oropa; **Event:** eventDate: 14.iv.2011; year: 2011; month: 4; day: 14; **Record Level:** institutionID: ZFMK; collectionID: MYR
- **Type status:** Other material. **Occurrence:** catalogNumber: 896; recordedBy: T. Wesener; individualID: TW07; individualCount: 1; sex: female; lifeStage: adult; **Taxon:** scientificName: Trachysphaera sp.; phylum: Arthropoda; class: Diplopoda; order: Glomerida; family: Glomeridae; genus: Trachysphaera; taxonRank: species; **Location:** locationID: 7; country: Italy; stateProvince: Piemonte; locality: Oropa, NW Sanctuary of Oropa; **Event:** eventDate: 14.iv.2011; year: 2011; month: 4; day: 14; **Record Level:** institutionID: ZFMK; collectionID: MYR

##### Notes

Additional individual information in Table 9.

## Analysis

### Molecular analysis

Despite differences in the morphology and coloration (see below), no variation in the mitochondrial haplotypes were found within *Trachysphaera* populations. Genetic distances (uncorr. p-dist.) in the COI sequence between the UK *Trachysphaera* populations and between *T. lobata* from France were low (1.9­–2.5%). The Isle of Wight *Trachysphaera* show a 2.5% divergence to the French population and a 1.9% genetic distance to the population from Wales. The Wales population shows an equal genetic distance of 1.9% to both the French and the Isle of Wight *Trachysphaera*.

All *T. lobata* populations show high genetic distances of 17.3–18.8% to the other *Trachysphaera* species from Italy and Croatia. The latter two, *T. schmidti* from Croatia and *T.* sp. from Italy, differ by 16.7%.

**Figure 3.**
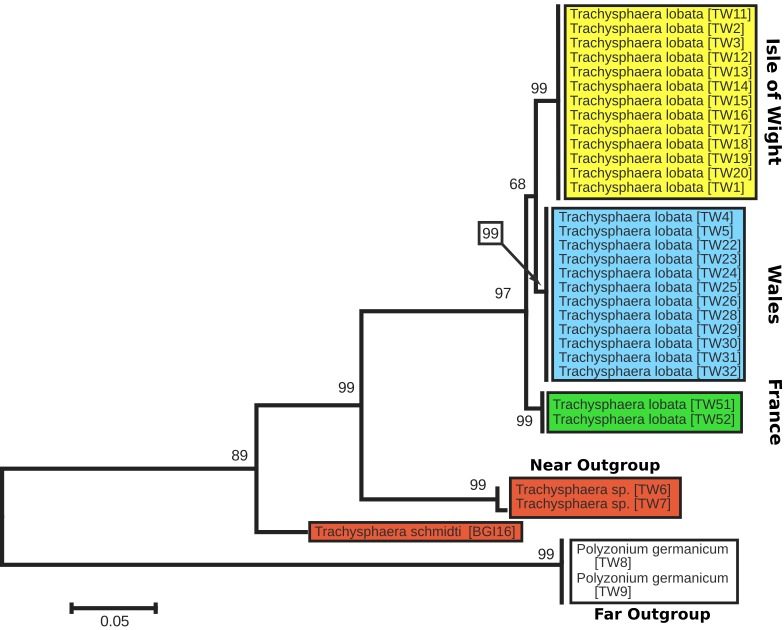
Maximum Likelihood Tree of 32 COI sequences of *Trachysphaera* and outgroup specimens. Rate model: discrete Gamma distribution, 5 categories (+*G*, parameter = 0.7759); nucleotide frequencies: A / T = 0.3273, C / G = 0.1727; tree is drawn to scale, branch lengths reflect number of substitutions per site; 1st, 2nd, 3rd codon positions and non-coding positions were included, gaps and missing data excluded; node values = 1000 replicates ML bootstrap support.

The close relationship of the UK and French *Trachysphaera* populations is also reflected in the phylogenetic tree (Fig. 3). The monophyly of *T. lobata* is well supported (97%), while the relationships between its three analyzed populations are less clear, with the sister-group of the Isle of Wight population only receiving moderate support of 68%. The Italian *Trachysphaera* population appears to be closer related to *T. lobata* (99% support) than to the type species of the genus, *T. schmidti*, despite a similar genetic distance (17.3% / 18.8%).

### Morphological character analysis - somatic characters

Most of the studied somatic characters show considerable variation (see Table 3), often independent of the observed generic variation (Fig. 4). Often, large variation in a character, such as number of toothed rows on the collum (Fig. 5a, c2), was observed even in the same specimen, with four rows on the left on five on the other side. Nevertheless, many characters were discovered to be constant between populations and species (Table 3). For example the endotergum (Fig. 5d, c5–7) displays no variation within the observed specimens.

The only unambiguous morphological characters that neither show great variation within a species, nor are constant between closely related species, are the characters 8–12 (Table 3). Characters 8 and 9 describe the number of rows of bacilli at the posterior margin of tergite 10 in the female and male sex, respectively. Here, *T. lobata* has two rows in both sexes (e.g., male: 852997, female: 853015), while *T. pyrenaica*, T. cf. rousseti and T. cf. drescoi have only a single row in both sexes (e.g., male: 852879, female: 852903). A second character, the presence of a protuberance on the anal shield (character 10 / 11 in Table 2), separates at least the males of *T. pyrenaica*, T. cf. drescoi and T. cf. rousseti from *T. lobata*, whose members lack such a conspicuous protuberance (e.g., present: 852829, absent: 852928).

### Morphological character analysis - the male telopods

The telopods of the studied *Trachysphaera* species show extensive variation between species, within species, within populations and even within the same individual (Figs 6, 7, 8). In *T. lobata* the male telopods show great differences in the shape of the femoral process, which is medially swollen in male TW14 (Fig. 6c), simply tapering in male TW12 (Fig. 6a) and with a special 'bump' in male TW21 (Fig. 7c), the latter being the only male specimen among the samples from Wales. Similar differences can be observed in the tibial process, which is barely visible in some *T. lobata* specimens from the Isle of Wight, but protruding in the single male from Wales (Fig. 7c, d). Even more pronounced are the differences between the left and right telopod of the *T. pyrenaica* male (GEU157). Here, the femoral process is triangular on the left telopod (Fig. 8c), while it is well-rounded, identical to those of *T. lobata* on the right telopod (Figs 6c, 8d). The differences between the left and right telopod are even more pronounced for the tibial process, which is well-developed and triangular on the left (Fig. 8c), but almost absent on the right telopod (Fig. 8d) of the same specimen.

## Discussion

### Interpretation of the results of the molecular analysis

The UK *Trachysphaera* populations belong, based on their mitochondrial DNA (COI sequence), clearly to *T. lobata*, an observation also corroborated by the morphological data (see below). Both populations analyzed (from the Isle of Wight and South Wales) show unique haplotypes.

Success of DNA extraction and PCR was generally low, indicating the necessity of special treatments to ensure success. Some specimens rolled-up so tight that ethanol was not able to penetrate the specimen, and muscle tissue was already partly decayed. We thus recommend to conserve specimens individually and open them shortly after conservation (i.e., a few minutes after death at best) so that ethanole can enter the organism. Furthermore, it is reasonable to not extract sequences from whole specimens but only from muscular and other tissues free of contamination from gut content or parasites. PCR success was improved (i.e., obtaining positive bands at all) with another deviation from standard protocols, namely using 5µl of DNA instead of the usual 1-2 µl. We were not able to study the reason for this necessity and can thus only speculate. Possibilities are insufficient primer matching, decay, or other, inherent peculiarities.

The differing results of sequencing attempts at the ZFMK and BGI despite of presumably standardized methods highlight the persistent difficulties of obtaining glomerid DNA sequences. Here, we deem a further investigation of best practice methods for Glomerida extremely important.

For studies of the intraspecific variation of *Trachysphaera* species, faster evolving markers that show a greater amount of variation should provide a better resolution than the COI gene fragment studied here. Unfortunately, such a marker is currently not available in Diplopoda.

Between *Trachysphaera* species, the genetic distance is quite high, on par with those observed between different genera of the order (see Wesener 2012). Potentially, the observable pattern of substitutions between the COI genes is already saturated. Here, more conservative markers like slowly evolving nuclear or ribosomal genes might allow more robust phylograms than provided. The large interspecific distances of the COI sequences (>15%) observed in different *Trachysphaera* species highlight the importance of this marker for barcoding purposes. Based on the available data, the barcoding gap is huge (­1.8–2.5% intraspecific vs. 15–­20% interspecific).

### Evaluation of morphological characters in *Trachysphaera*

Telopods need to be studied and illustrated with great care. Slight variations of the angle of view can provide very different observations (compare Figs 6, 7, 8). Additionally, vast differences of telopod shape do not correspond to observed genetic differences. Thus, we deem telopods no good character of trachysphaeran species discrimination, despite their use in other studies.

Since trachysphaeran surface structures are tiny, complex, and manifold, their evaluation as well as drawing conclusions is obfuscated. Many characters are too fine-scaled or complex to be evaluated in a discrete fashion and it is hard to define categories due to variability in quasi-countable characters. The possible inner variation of extended structures (e.g., endotergum) confounds decisions of species identification based on small details of the structure. However, even small details vary in their peculiarities: large sclerotized protuberances (LSPs; Fig. 5a, c4) form three rows in the middle of the thoracic shield (TS) but dissolve into swirls on the edges of the TS. This means, defining characters as well as states is problematic. The only character sets that seem to allow the discrimination of *T. lobata* from the rest of the species in our taxon sample are the number of rows of bacilli at the posterior margin of tergite 10 and the shape of the anal shield (characters 8-12 in Table 3). Examples for these are depicted in Fig. 9, using males as they show the features more pronounced.

Problems did not only arise from the structural diversity itself but also from the method of documentation. Too few pictures were taken and not all angles were available due to fixation. Comparing all specimens, not all structures were shot from the same angle. A better chance to find true species-specific characters and evaluate them properly would be given by zoomable 3D images, as can be obtained by micro-CT studies. Additionally, physical influences may have distorted the evaluation. LSPs and similar extended structures (e.g., setae) especially may have been evaluated inconsistently due to prior abrasion (during struggle in life, dissection, mounting and sputtering). Grooves may have been evaluated incorrectly if clogged by dirt. Here, testing whether ultra-sonic cleaning is feasible would be beneficial (we did not try due to the risk of loosing of one specimen). Given these complications and our relatively small sample size, further corroboration of our results is desirable. Meanwhile, our conclusions have to be considered as preliminary, but nevertheless relevant.

### The origin and identity of the British *Trachysphaera* populations

Both, barcoding and the morphological study identify the British *Trachysphaera* populations as *T. lobata*. We were not able to trace the origin of these British populations because only one French mainland population could be sampled due to external circumstances. However, an independent origin of the South Wales and the Isle of Wight populations could be ascertained. Whether this independent origin is the result of two different recent anthropogenic introduction events or if they represent two relic populations of a once widespread occurrence currently cannot be determined. Once the COI sequence of more French populations of *T. lobata* becomes known, a clearer picture of the origins of the British *Trachysphaera* can be drawn.

## Supplementary Material

XML Treatment for Trachysphaera lobata

XML Treatment for Trachysphaera pyrenaica

XML Treatment for Trachysphaera rousseti

XML Treatment for Trachysphaera drescoi

XML Treatment for Trachysphaera schmidti

XML Treatment for Trachysphaera sp.

## Figures and Tables

**Figure 1a.**
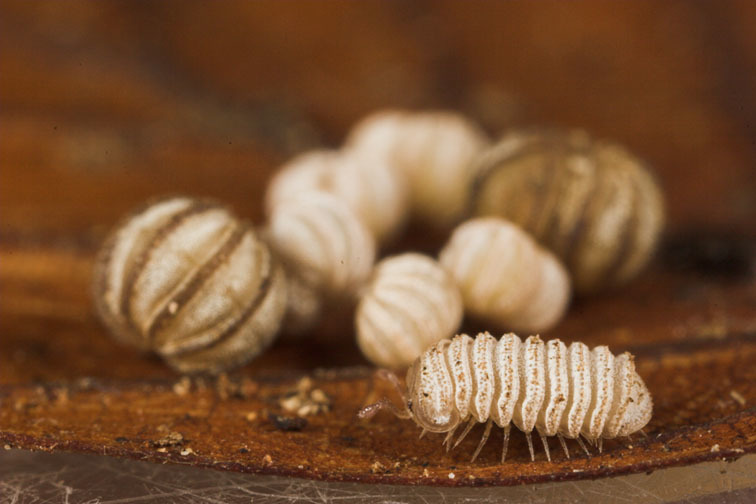


**Figure 1b.**
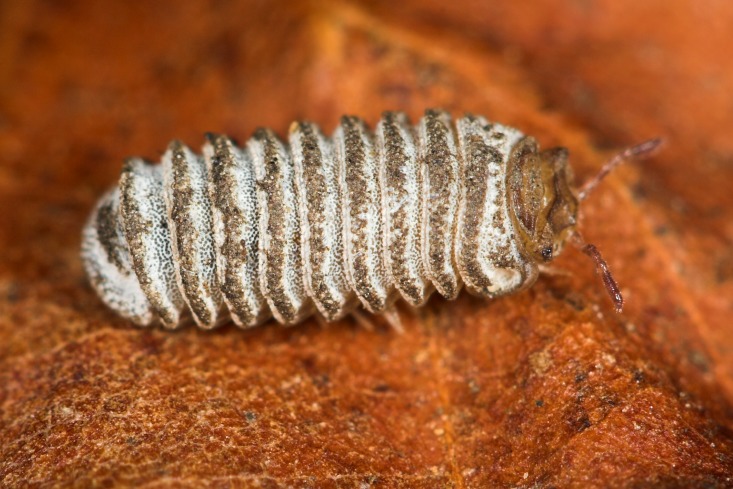


**Figure 2a.**
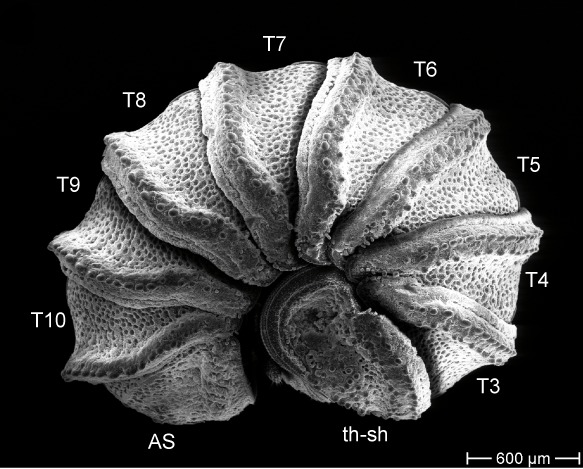
lateral view, showing slightly unrolled specimen

**Figure 2b.**
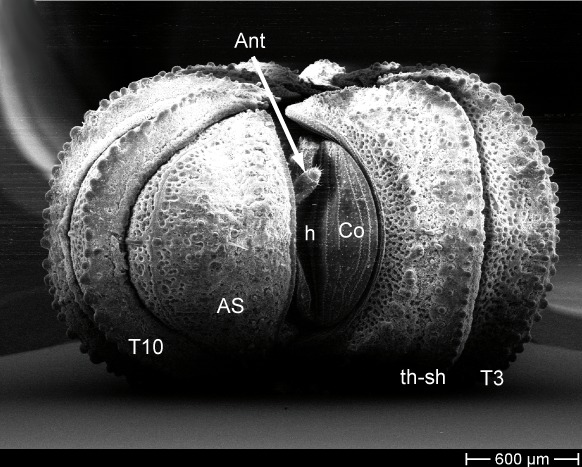
same specimen, view of the opening between anal shield and thoracic shield, exposing head and collum (reduced first tergite) usually held inside the sphere when rolled up

**Figure 4a.**
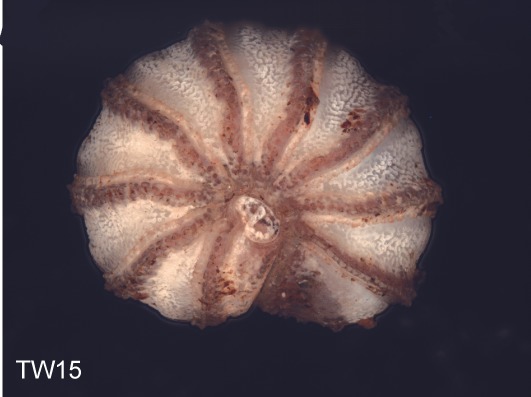
TW15: *T. lobata*, male, Isle of Wight

**Figure 4b.**
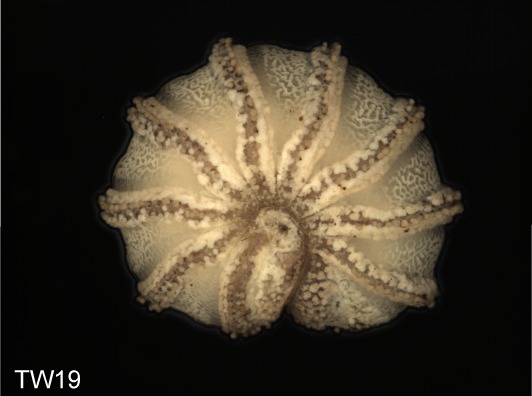
TW18: *T. lobata*, female, Isle of Wight

**Figure 4c.**
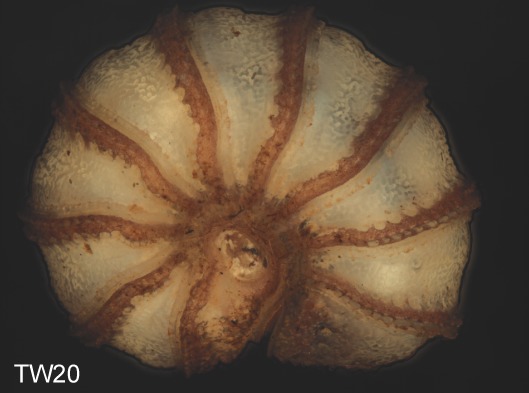
TW20: *T. lobata*, male, Isle of Wight

**Figure 4d.**
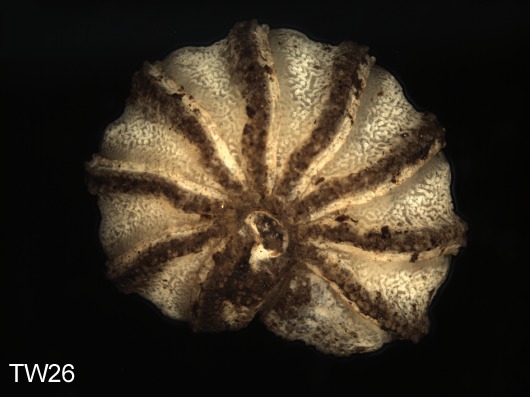
TW26: *T. lobata*, female, South Wales

**Figure 4e.**
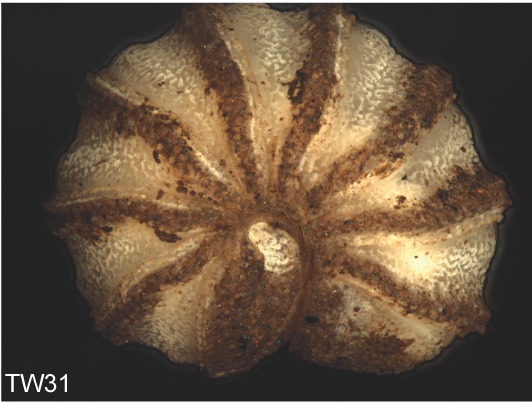
TW31: *T. lobata*, female, South Wales

**Figure 4f.**
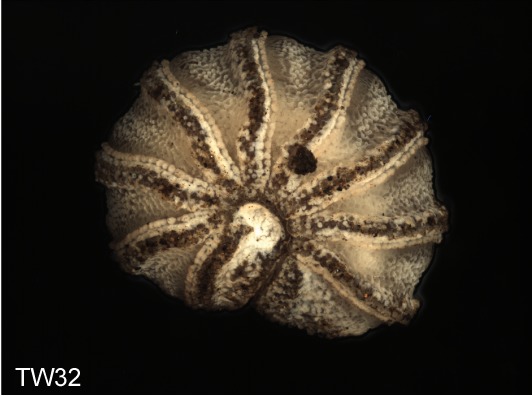
TW32: *T. lobata*, female, South Wales

**Figure 5a.**
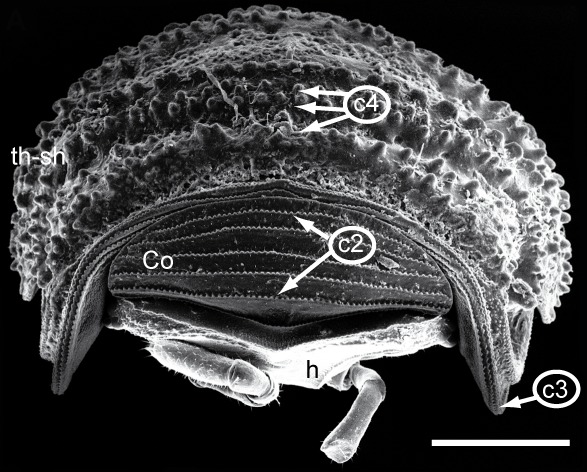
TW1: *T. lobata*, female, Isle of Wight; anterior view; scale bar: 400 µm

**Figure 5b.**
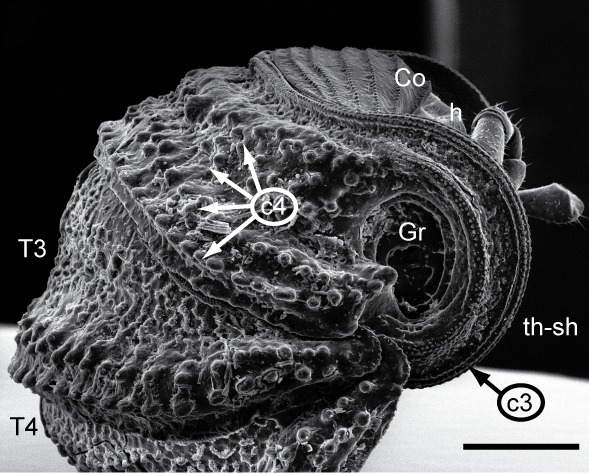
TW1: *T. lobata*, female, Isle of Wight; anterior body part, lateral view; scale bar: 300 µm

**Figure 5c.**
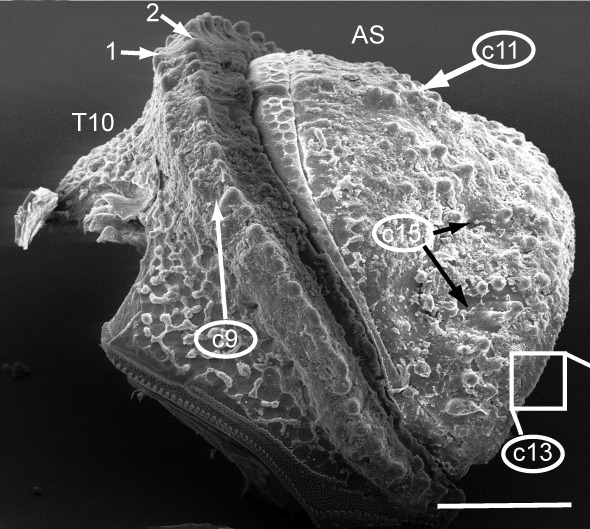
TW29: *T. lobata*, female, South Wales; tergite 10 and anal shield, lateral view; scale bar: 300 µm; c13 points to Fig. 4e

**Figure 5d.**
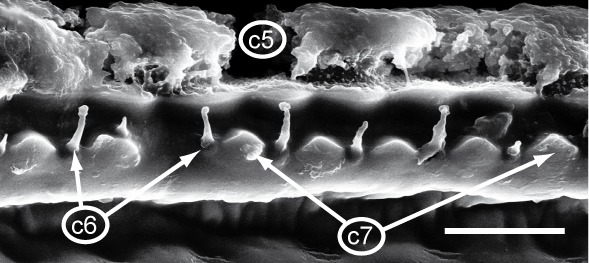
TW29: *T. lobata*, female, South Wales; endotergum (underside of tergite); scale bar: 20 µm

**Figure 5e.**
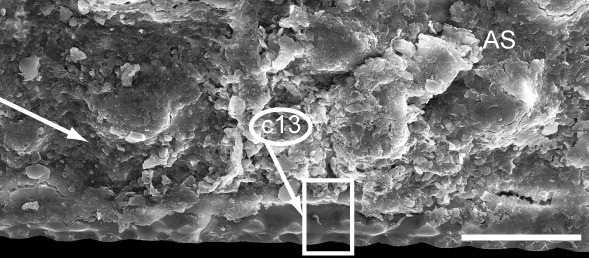
TW29: *T. lobata*, female, South Wales; posterior margin of anal shield, detail; scale bar: 50 µm

**Figure 6a.**
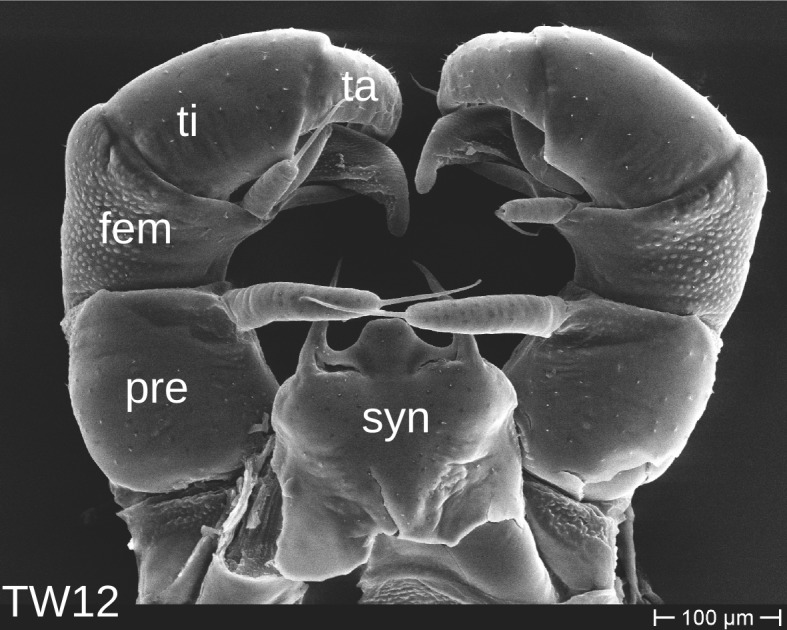
anterior view (MBiID 852943)

**Figure 6b.**
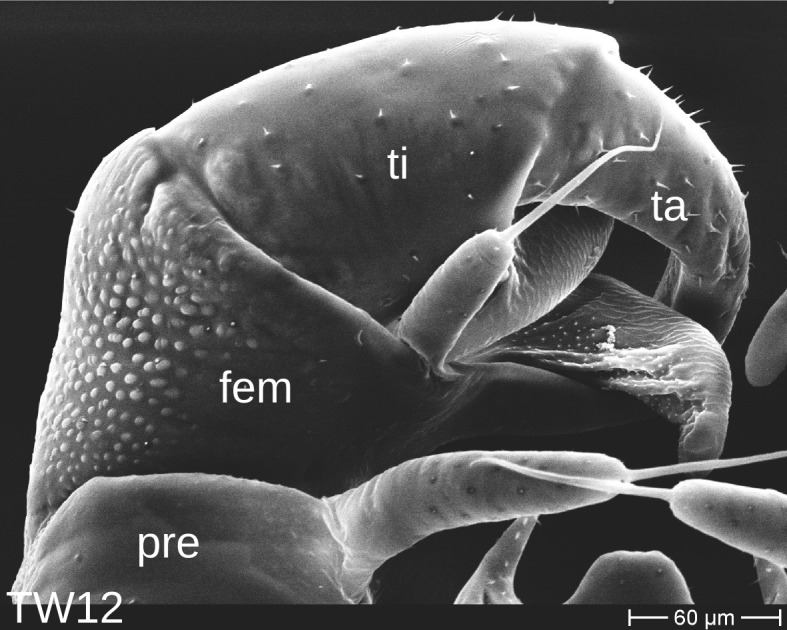
anterior view (MBiID 852942)

**Figure 6c.**
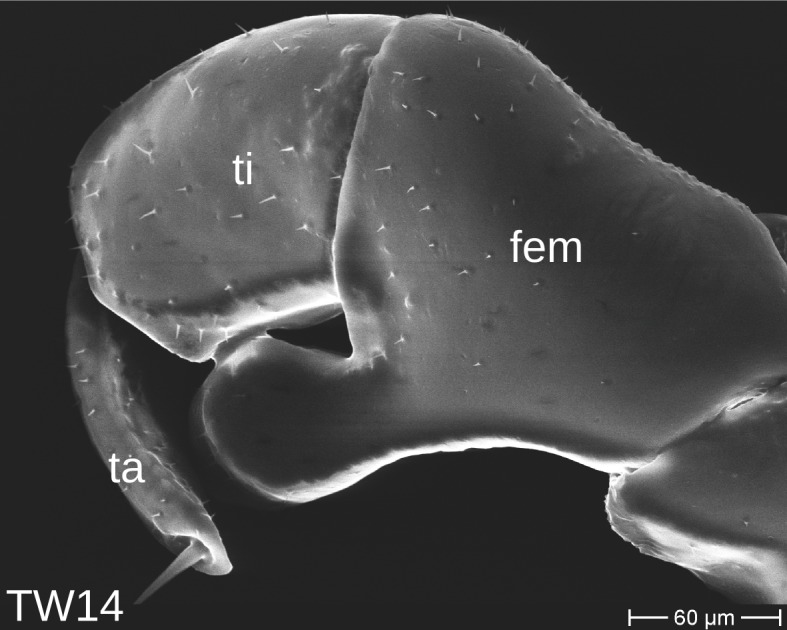
posterior (anal) view (MBiID 852974)

**Figure 6d.**
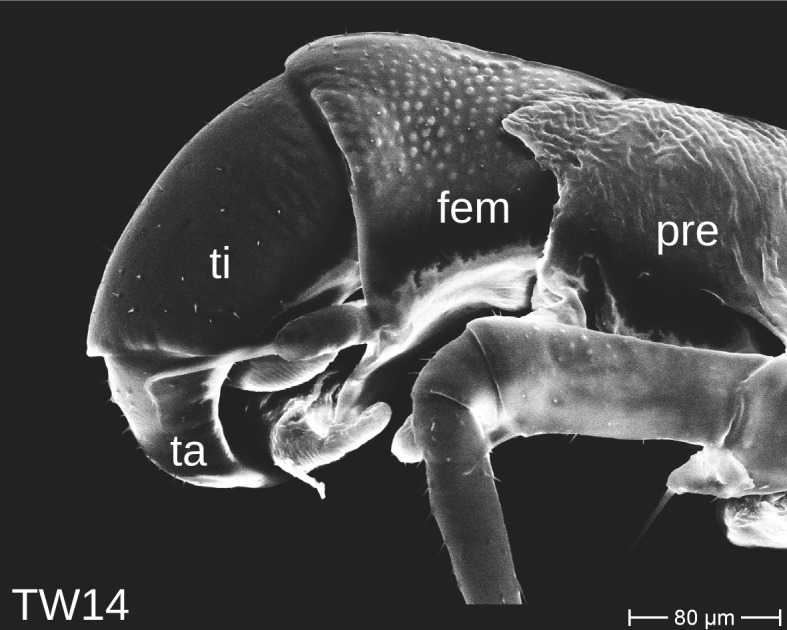
anterior view (MBiID 852977)

**Figure 7a.**
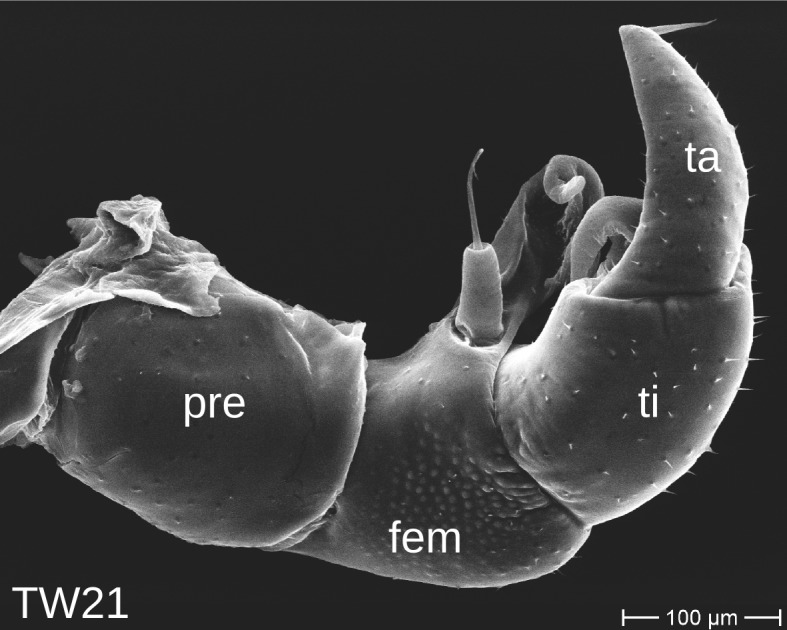
anterior view (MBiID 853010)

**Figure 7b.**
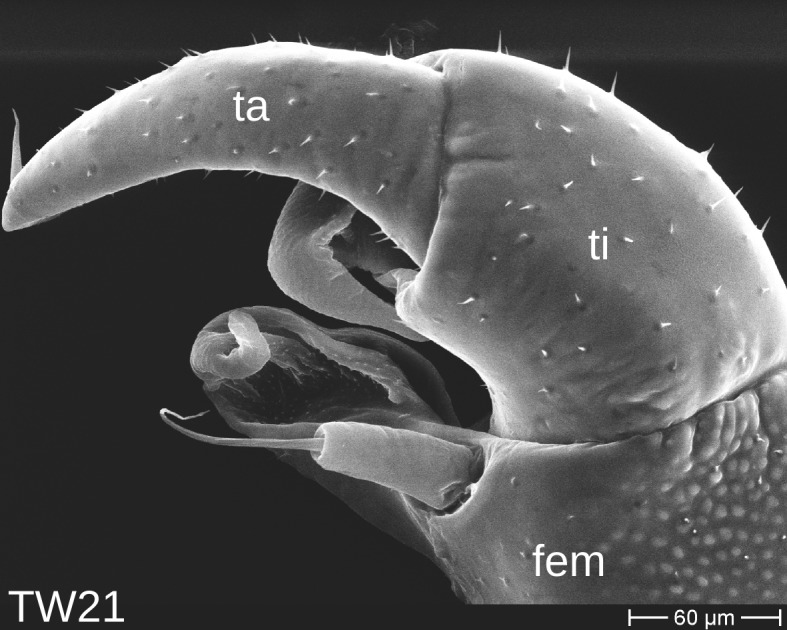
anterior view (MBiID 853007)

**Figure 7c.**
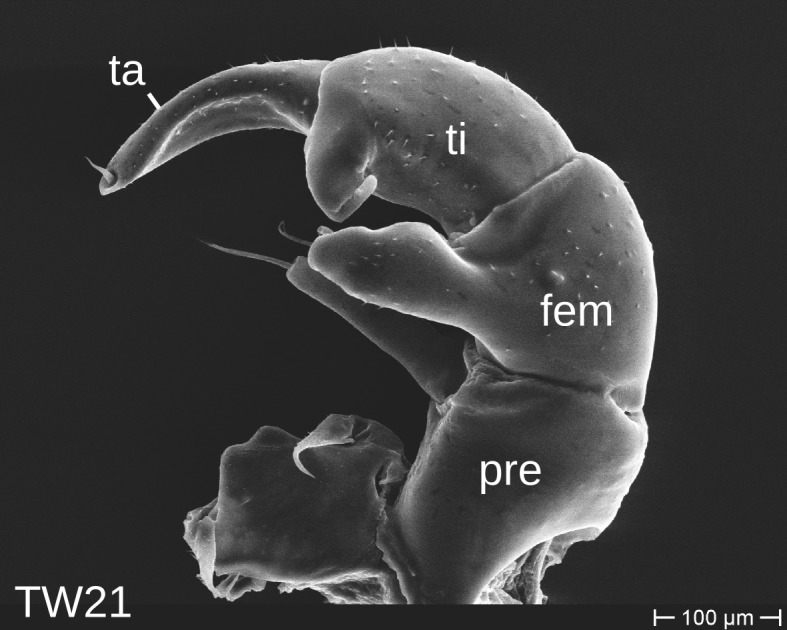
posterior (anal) view (MBiID 853009)

**Figure 7d.**
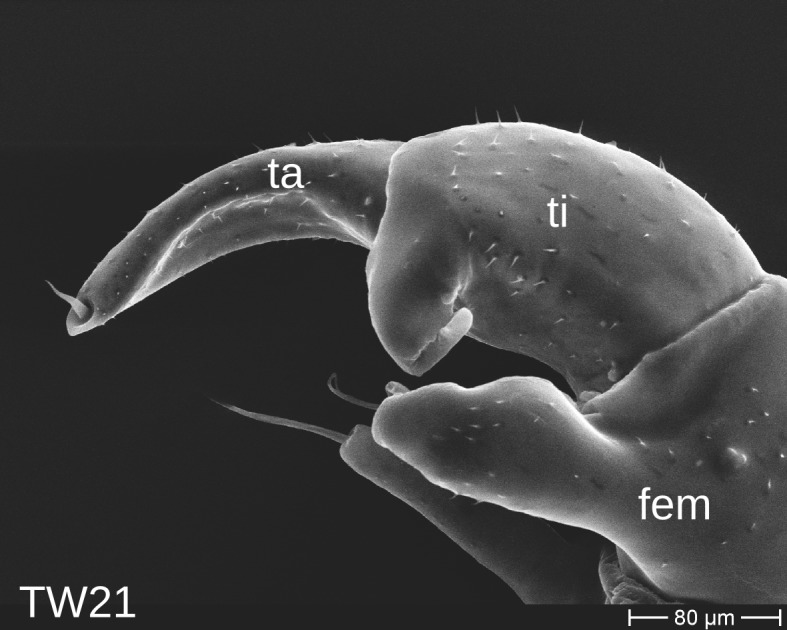
posterior (anal) view (MBiID 853008)

**Figure 8a.**
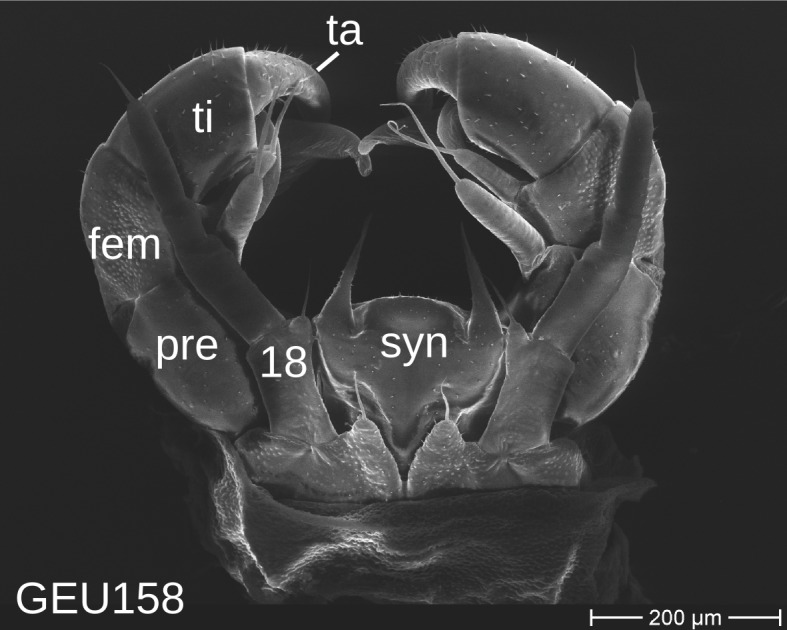
T. cf. drescoi, anterior view (MBiID 852849)

**Figure 8b.**
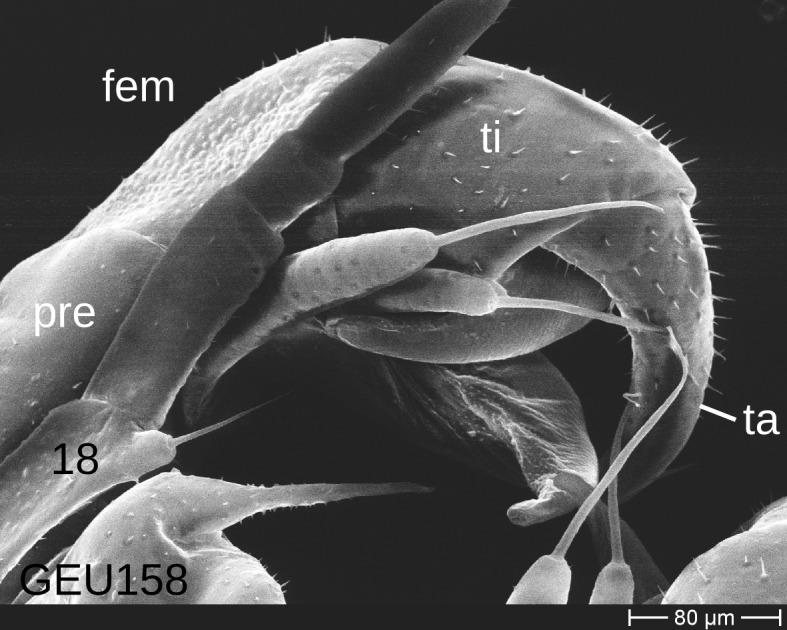
T. cf. drescoi, anterior view (MBiID 852848)

**Figure 8c.**
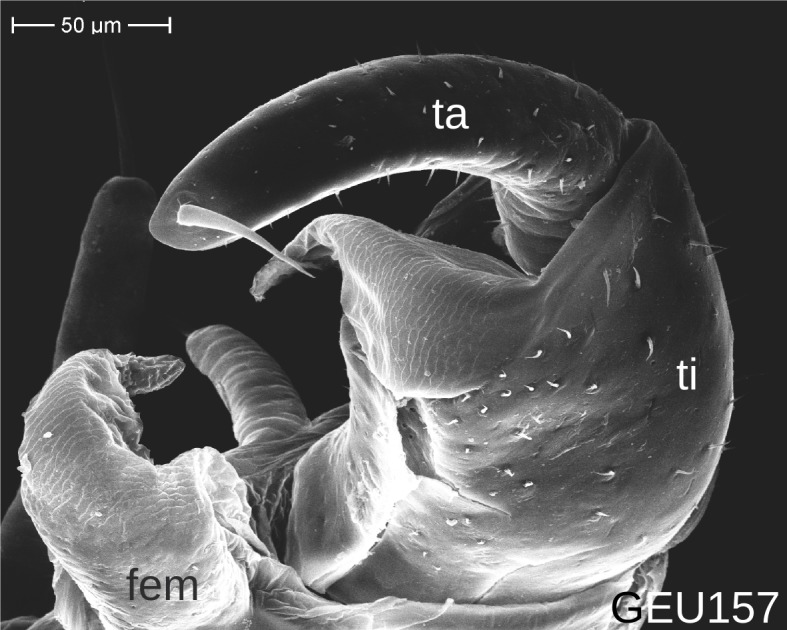
*T. pyrenaica*, anterior view (MBiID 852894)

**Figure 8d.**
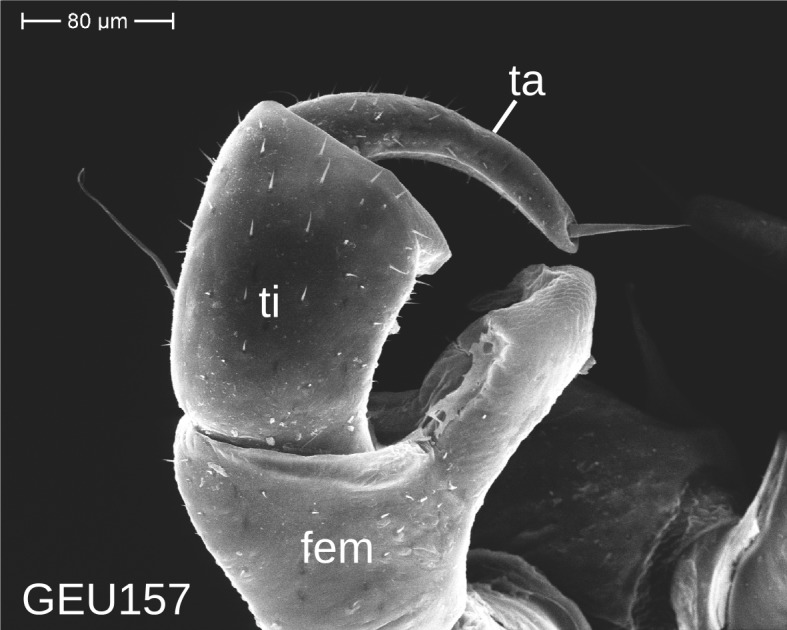
*T. pyrenaica*, posterior (anal) view (MBiID 852898)

**Figure 9a.**
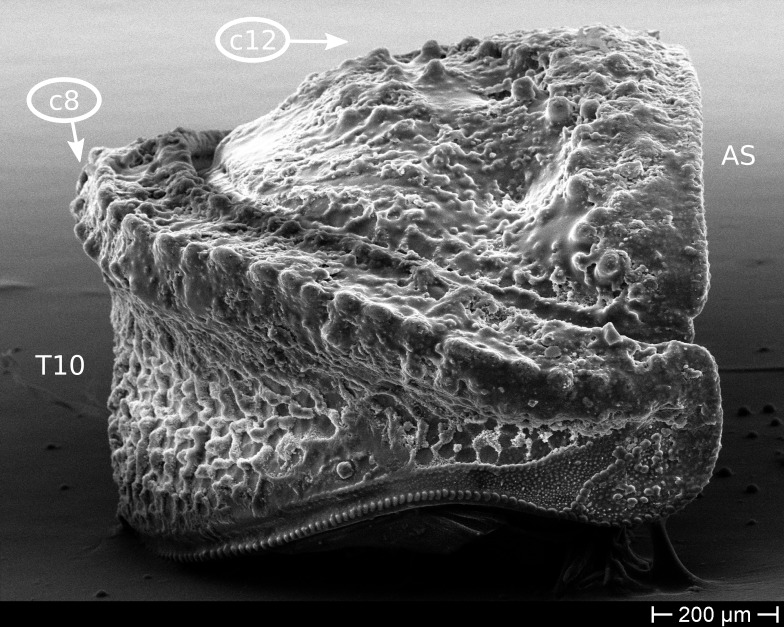
TW30: *T. lobata*, male, South Wales (MBsID: 852824). Character states: two rows of bacilli on posterior edge of T10 (c8/1) and well-rounded anal shield (c12/0). This set of character states is (in our sample) unique for *T. lobata*.

**Figure 9b.**
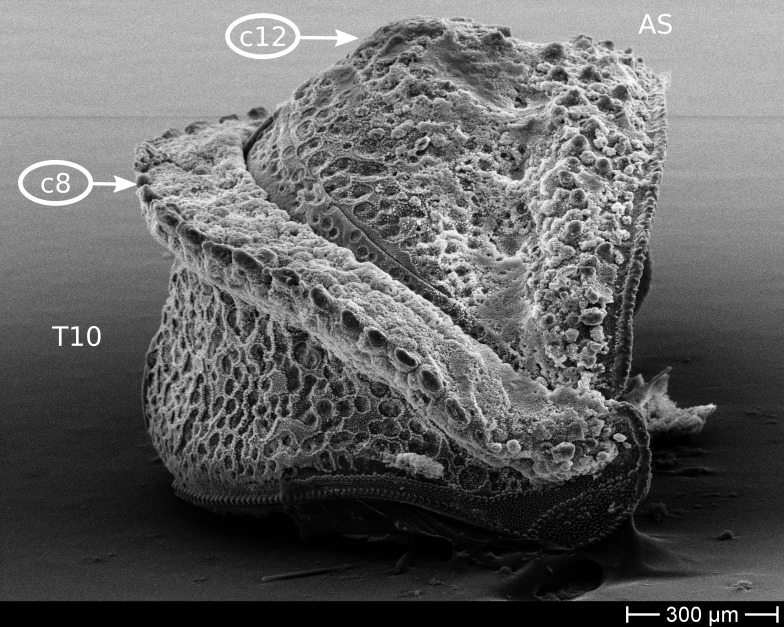
GL07: T. cf. rousseti, male, Spain (MBsID: 852825). Character states: one row of bacilli on posterior edge of T10 (c8/0) and anal shield with special protuberance (c12/1). This set of character states is (in our sample) shared by *T. pyrenaica*, T. cf. rousseti, and T. cf. drescoi.

**Table 1.** Localities and method application. Locality ID [LocID] as given in Checklist Materials, Extracted specimens [# Extracted], PCR and sequencing success [# PCR success, # Sequencing success] and number of *Trachysphaera* specimens studied under SEM [# SEM]. Numbers in parentheses refer to specimens where PCR and/or sequencing were unsuccessful at the ZFMK but successful at the BGI. More detailed locality info in Checklist section.

**LocID**	**Locality**	**# Extracted**	**# PCR success**	**# Sequencing success**	**# SEM**
1	Isle of Wight, GB	13	13	13	5
2	South Wales, GB	15	15	12	8
3	Génis, FR	10	4 (2)	- (2)	-
4	Grotte de l'Estellas, FR	1	-	-	2
5	Leitza, ESP	1	-	-	1
6	Sare, FR	1	-	-	2
7	Oropa, IT	2	2	2	-
8	Velika-Kapela, CRO	1	- (1)	- (1)	-

**Table 2.** Characters and states. Character numbers [C #], states are exemplified with SEM images partly in figures, partly with links to MorphBank, then given as MBiIDs.

**C #**	**Character**	**States**
**1**	Color of freshly preserved specimen.	(0) brownish (Fig. 4f), or (1) whitish (Fig. 4b)
**2**	Collum (reduced tergite 1), number of toothed ridges (Fig. 5a).	(0) 4/5 (853103), (1) 5 (852868), (2) 5/6 (852842)
**3**	Thoracic shield (enlarged tergite 2) anterior margin, number of rows of sclerotized nodules (Fig. 5a, b).	Real number
**4**	Thoracic shield, number of rows of large sclerotized protuberances (Fig. 5a, b).	Real number
**5**	Endotergum (underside of posterior margin of tergites), structure.	(0) simple margin with one row of sclerotized nodules, and single row of short setae (Fig. 5d, 852889), (1) more complex (no example found)
**6**	Endotergum, number of rows of setae.	(0) 1 (Fig. 5d), (1) >1 (no example found)
**7**	Endotergum, number of rows of sclerotized nodules.	(0) 1 (Fig. 5d), (1) >1 (no example found)
**8**	Male tergite 10, posterior margin, number of rows of large bacilli.	(0) 1 (852885), (1) 2 (852928)
**9**	Female tergite 10, posterior margin, number of rows of large bacilli (Fig. 5c).	(0) 1 (852903), (1) 2 (853068)
**10**	Male anal shield, shape.	(0) well-rounded (852928), (1) with special protuberance (852829)
**11**	Female anal shield, shape (Fig. 5c).	0) well-rounded (852960), (1) with special protuberance (852856)
**12**	Male anal shield, setae at posterior margin.	(0) isolated, 1-2 rows, (852978), (1) many, >2 rows (852826)
**13**	Female anal shield, setae at posterior margin (Fig. 5c, e).	(0) isolated, 1-2 rows, (853011), (1) many, >2 rows (853027)
**14**	Male anal shield, large circular grooves.	(0) absent or hard to see (852885), (1) prominent (852827)
**15**	Female anal shield, large circular grooves (Fig. 5c).	(0) absent or hard to see (853080), (1) prominent (853139)

**Table 3.** Character matrix. **Abbreviations**: [N] refers to number of individuals investigated by SEM; IoW = Isle of Wight population; U = character conflict within one population, i.e., unresolved.

**Species/Population**	**1**	**2**	**3**	**4**	**5**	**6**	**7**	**8**	**9**	**10**	**11**	**12**	**13**	**14**	**15**
*T. pyrenaica* [N=2]	**U**	0	0	1	0	0	0	0	0	1	0	0	0	0	0
T. cf. rousseti [N=1]	1	0	0	1	0	0	0	n/a	0	n/a	1	n/a	0	n/a	1
T. cf. drescoi [N=2]	**U**	**U**	0	**U**	0	0	0	0	0	1	1	1	1	1	1
*T. lobata* IoW [N=5]	**U**	0	0	**U**	0	0	0	1	1	0	0	0	**U**	**U**	**U**
*T. lobata* Wales [N=8]	**U**	**U**	0	**U**	0	0	0	1	1	0	0	**U**	**U**	**U**	**U**

**Table 4.** Additional sample information for *Trachysphaera lobata* specimens. Material specimen number as used above, Extraction voucher number, MorphBank specimen ID (with the number of SEM images available in parentheses), NCBI GenBank accession number.

Material specimen #	Extraction voucher #	MBspecimenID	GenBank accession #
a	TW01	852812 (15)	KJ408484
b	TW02	–	KJ408485
c	TW03	–	KJ408486
d	TW04	–	KJ408497
e	TW05	–	KJ408498
f	TW11	–	KJ408487
g	TW12	852813 (17)	KJ408488
h	TW13	852814 (11)	KJ408489
i	TW14	852815 (23)	KJ408490
j	TW15	–	KJ408491
k	TW16	–	KJ408492
l	TW17	852816 (17)	KJ408493
m	TW18	–	KJ408494
n	TW19	–	KJ408495
o	TW20	–	KJ408496
p	TW21	852817 (16)	–
q	TW22	852818 (16)	KJ408499
r	TW23	–	KJ408500
s	TW24	852819 (21)	KJ408501
t	TW25	852820 (18)	KJ408502
u	TW26	852821 (12)	KJ408503
v	TW27	852822 (13)	–
w	TW28	–	KJ408504
x	TW29	852823 (28)	KJ408505
y	TW30	852824 (17)	KJ408506
z	TW31	–	KJ408507
aa	TW32	–	KJ408508
ab	TW33	–	–
ac	TW47	–	–
ad	TW48	–	–
ae	TW49	–	–
af	TW50	–	–
ag	TW51	–	KJ408482
ah	TW52	–	KJ408483
ai	TW53	–	–
aj	TW54	–	–
ak	TW55	–	–
al	TW57	–	–

**Table 5.** Additional sample information for *Trachysphaera pyrenaica* specimens. Material specimen number as used above, Extraction voucher number, MorphBank specimen ID (with the number of SEM images available in parentheses), NCBI GenBank accession number.

Material specimen #	Extraction voucher #	MBspecimenID	GenBank accession #
a	GEU157	852809 (7)	–
b	GEU157	852810 (23)	–

**Table 6.** Additional sample information for Trachysphaera cf. rousseti specimen. Material specimen number as used above, Extraction voucher number, MorphBank specimen ID (with the number of SEM images available in parentheses), NCBI GenBank accession number.

Material specimen #	Extraction voucher #	MBspecimenID	GenBank accession #
a	GL017	852825 (16)	–

**Table 7.** Additional sample information for Trachysphaera cf. drescoi specimens. Material specimen number as used above, Extraction voucher number, MorphBank specimen ID (with the number of SEM images available in parentheses), NCBI GenBank accession number.

Material specimen #	Extraction voucher #	MBspecimenID	GenBank accession #
a	GEU158	852808 (20)	–
b	GEU158	852807 (24)	–

**Table 8.** Additional sample information for *Trachysphaera schmidti* specimen. Material specimen number as used above, Extraction voucher number, MorphBank specimen ID (with the number of SEM images available in parentheses), NCBI GenBank accession number.

Material specimen #	Extraction voucher #	MBspecimenID	GenBank accession #
a	BGI-MYR-16	–	KJ408481

**Table 9.** Additional sample information for *Trachysphaera* sp. specimens. Material specimen number as used above, Extraction voucher number, MorphBank specimen ID (with the number of SEM images available in parentheses), NCBI GenBank accession number.

Material specimen #	Extraction voucher #	MBspecimenID	GenBank accession #
a	TW06	–	–
b	TW07	–	KJ408480
